# Supplementary material for: The Synthesis, Characterization, and Fluxional Behavior of a Hydridorhodatetraborane
Source: Molecules. 2023 Sep 6;28(18):6462. doi: 10.3390/molecules28186462 (PMC10535517; doi:10.3390/molecules28186462)
Supplement: Supplementary file 1 [file molecules-28-06462-s001.zip › molecules-2550281-supplementary.pdf]

The Synthesis, Characterization, and Fluxional Behavior of a Hydridorhodatetraborane

Fatou Diaw-Ndiaye, Pablo J. Sanz Miguel, Ricardo Rodríguez and Ramón Macías

Departamento de Química Inorgánica, Instituto de Síntesis Química y Catálisis Homogénea (ISQCH), Universidad de Zaragoza-CSIC, 50009 Zaragoza, Spain

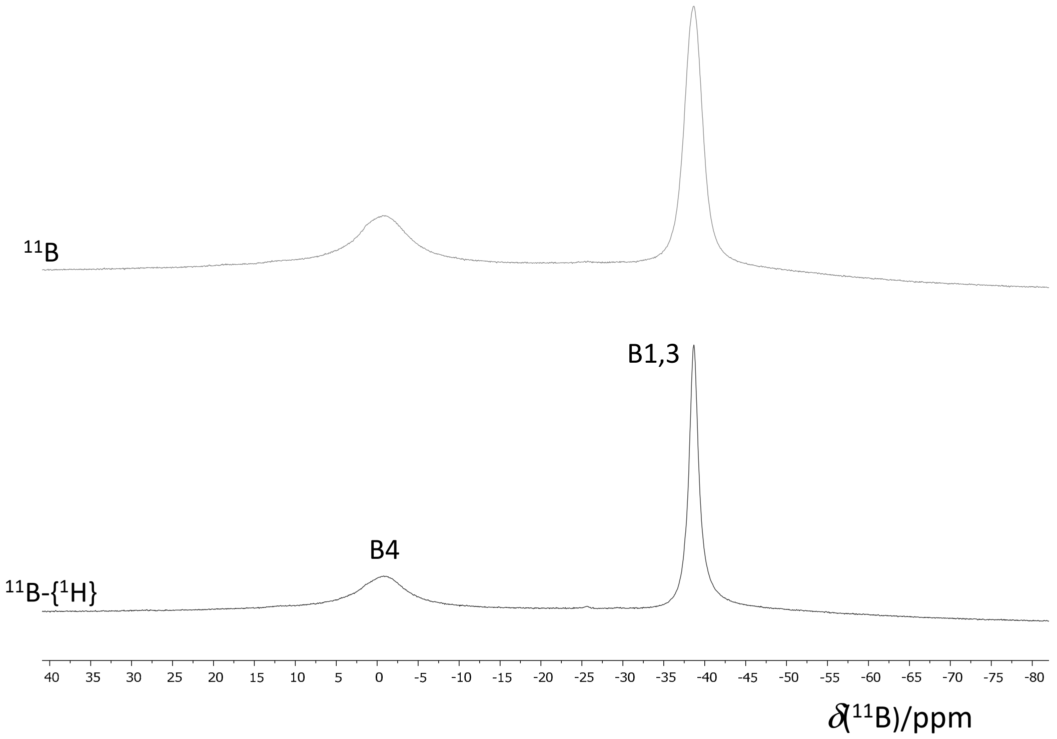

Figure S1 ^{11}B-{^{1}H} and ^{11}B NMR spectra of **1** in CD_2Cl_2, at 298 K, 96 MHz.

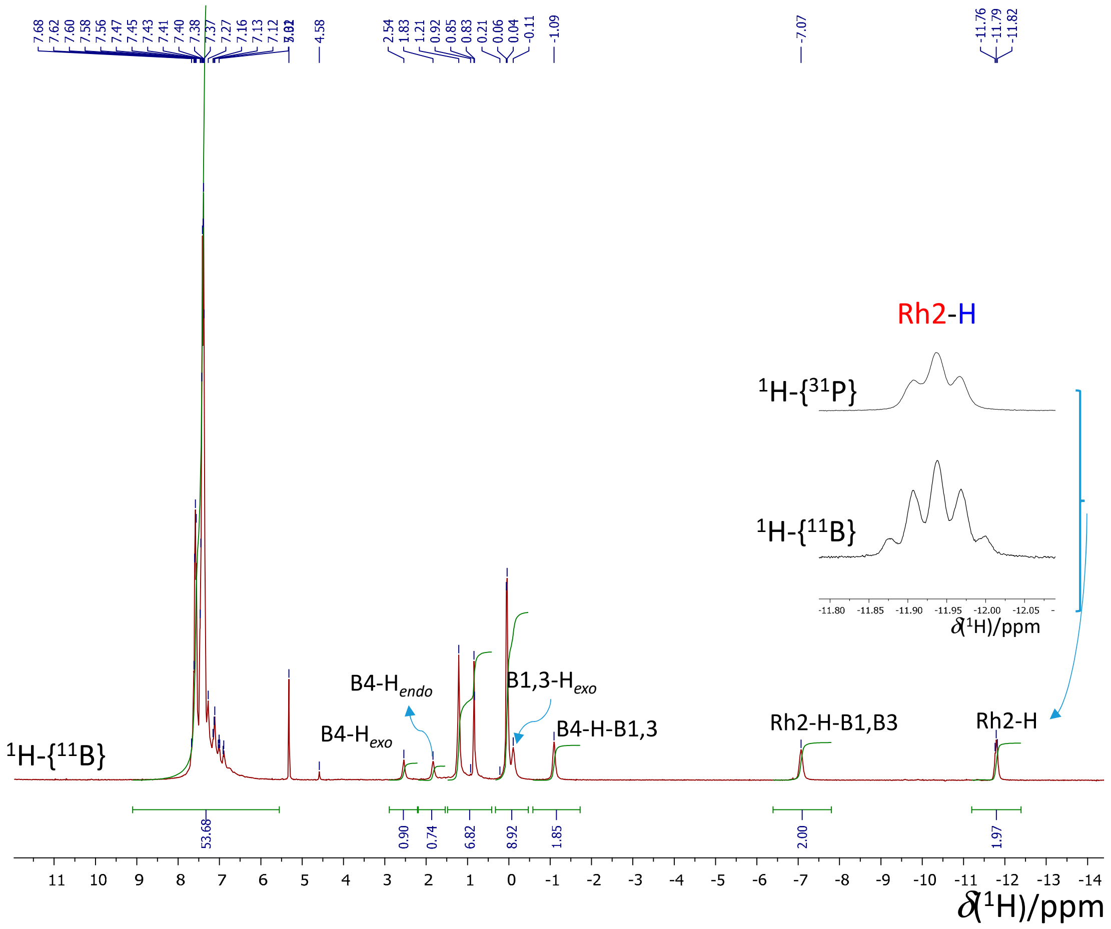

Figure S2 500 MHz, ^{1}H–{^{11}B} NMR spectrum (red trace) at 223 K, in CD$_2$Cl$_2$; and inlet showing the Rh2–H signal upon boron-11 and phosphorous-31 decoupling.

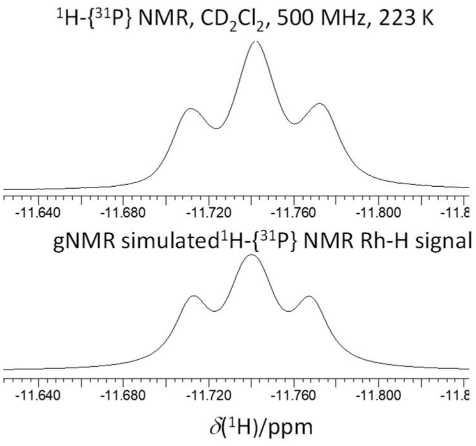

	Nucleus Name	n	δ ppm	W Hz	J 1	2	3	4
1	1H	1	-7.029	20.00				
			–	–				
2	1H	1	-7.029	20.00	0.00			
			–	–	–			
3	1H	1	-11.740	9.00	2.00	12.00		
			–	–	–	–		
4	1H	1	-11.740	9.00	12.00	2.00	0.00	
			–	–	–	–	–	
5	103Rh	1	0.000		18.00	18.00	16.00	16.00

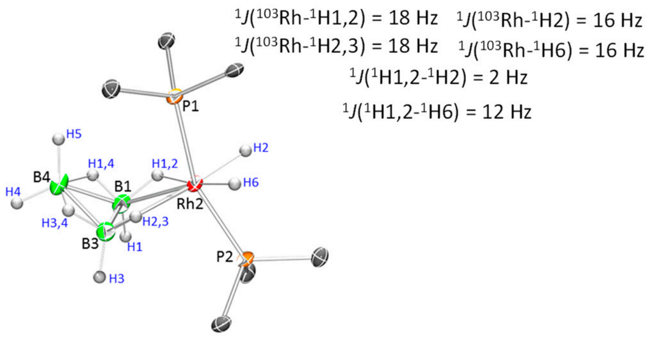

Figure S3 Simulation of the Rh$_2$–H hydride signal in the 1H-{31P} spectrum of [Rh(^{2}-B$_3$H$_8$)(H)$_2$(PPh$_3$)$_2$] (**1**), in CD2Cl$_2$.

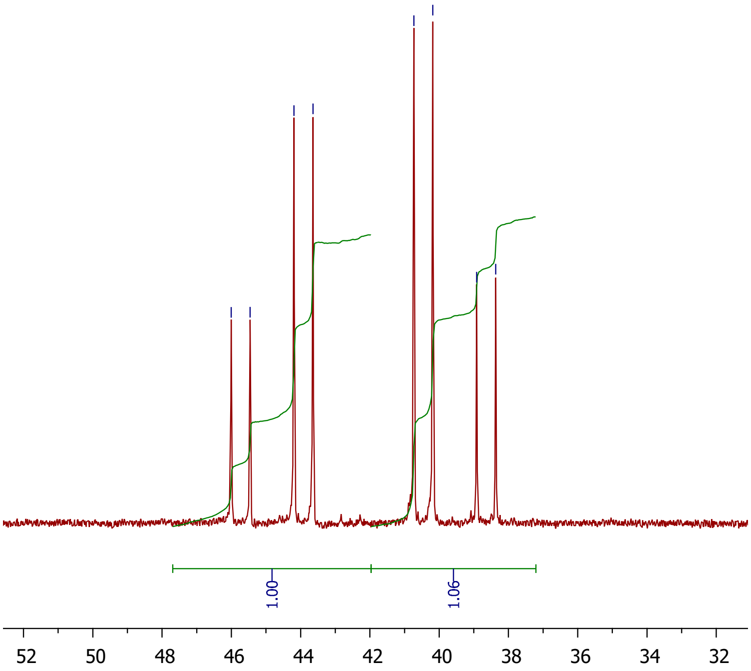

Figure S4 ^{31}P-{^{1}H} NMR spectra of $[Rh(\ ^{2}\text{-}B_3H_8)(H)_2(PPh_3)_2]$ (**1**), in CD_2Cl_2, at 223 K.

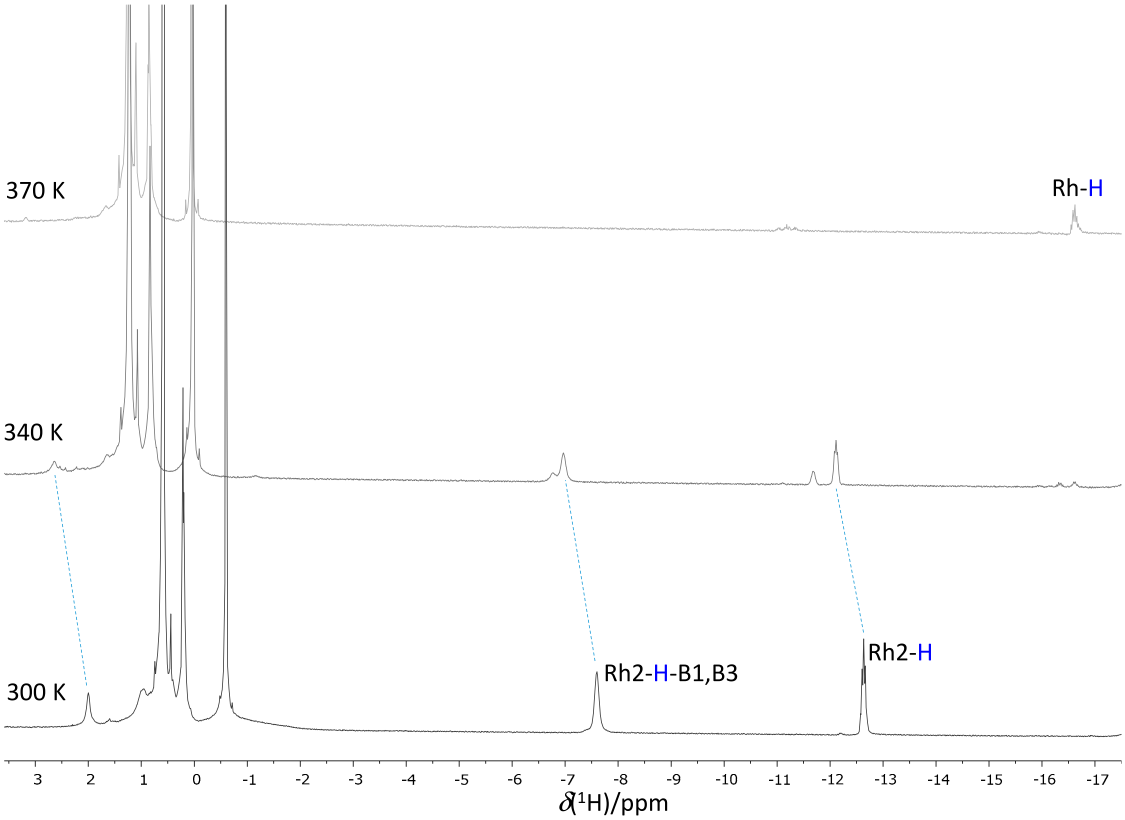

Figure S5 ^{1}H-{^{11}B} NMR spectra of $[Rh(\ ^{2}\text{-}B_3H_8)(H)_2(PPh_3)_2]$ (**1**), in $Cl_2DC–CDCl_2$, as function of the temperature.

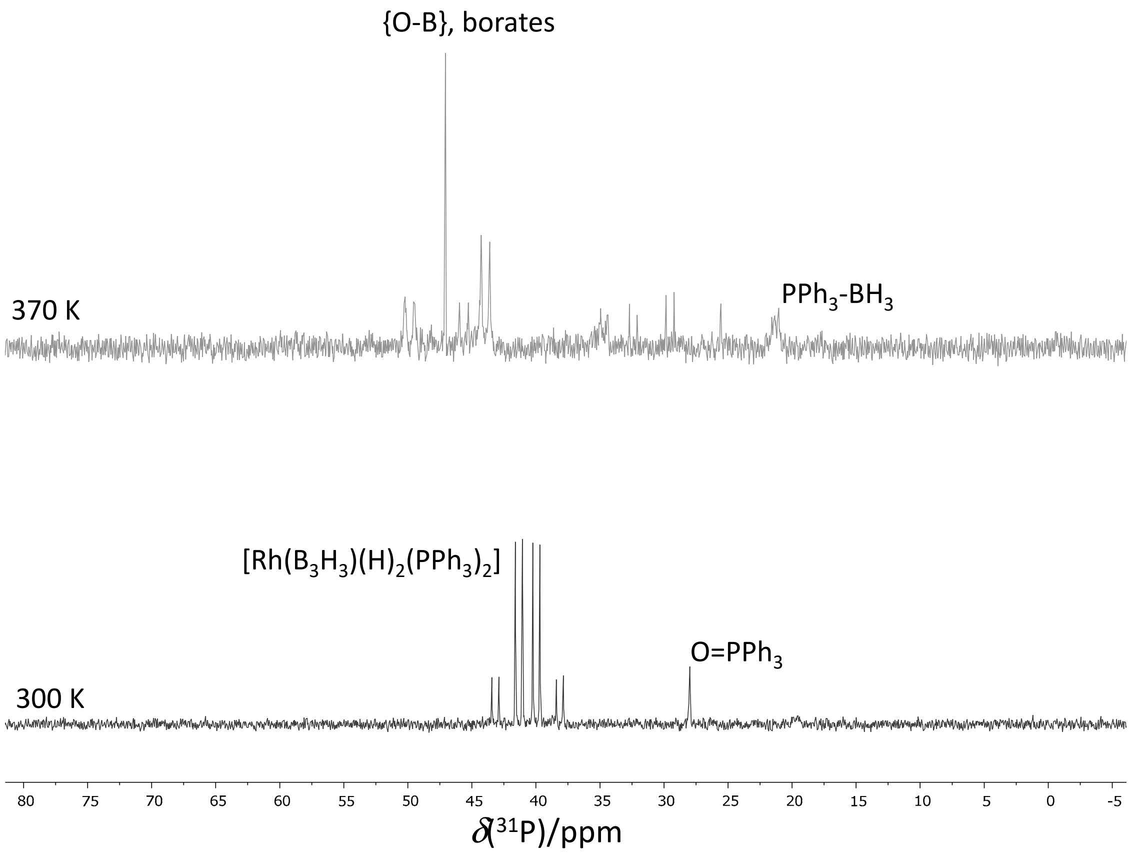

Figure S6 ^{31}P-{^{1}H} NMR spectra of [Rh(^{2}-B_3H_8)(H)$_2$(PPh_3)$_2$] (**1**), in $Cl_2DC–CDCl_2$, as function of the temperature:

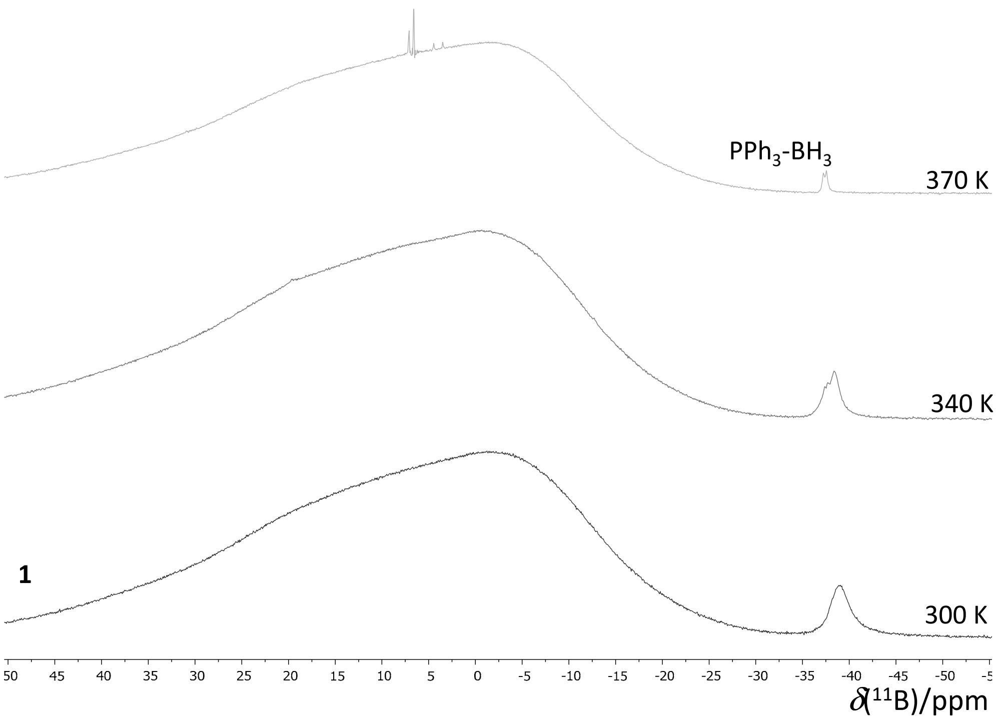

Figure S7 ^{11}B-{^{1}H} NMR spectra of [Rh(^{2}-B_3H_8)(H)$_2$(PPh_3)$_2$] (**1**), in $Cl_2DC–CDCl_2$, as function of the temperature: decomposition to give borates and the *tris*-phenylphosphine adduct.

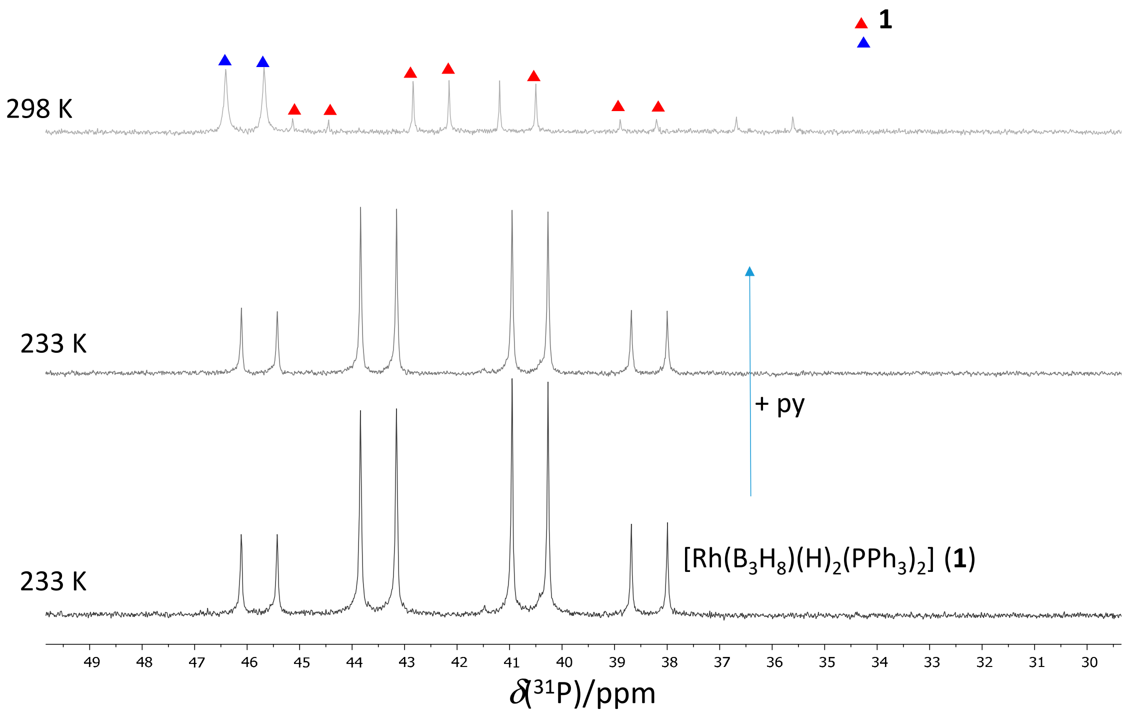

Figure S8 ^{31}P-{^{1}H} NMR spectra from the reaction between [Rh(^{2}-B_3H_8)(H)$_2$(PPh$_3$)$_2$] (**1**) and pyridine, in dichloromethane.

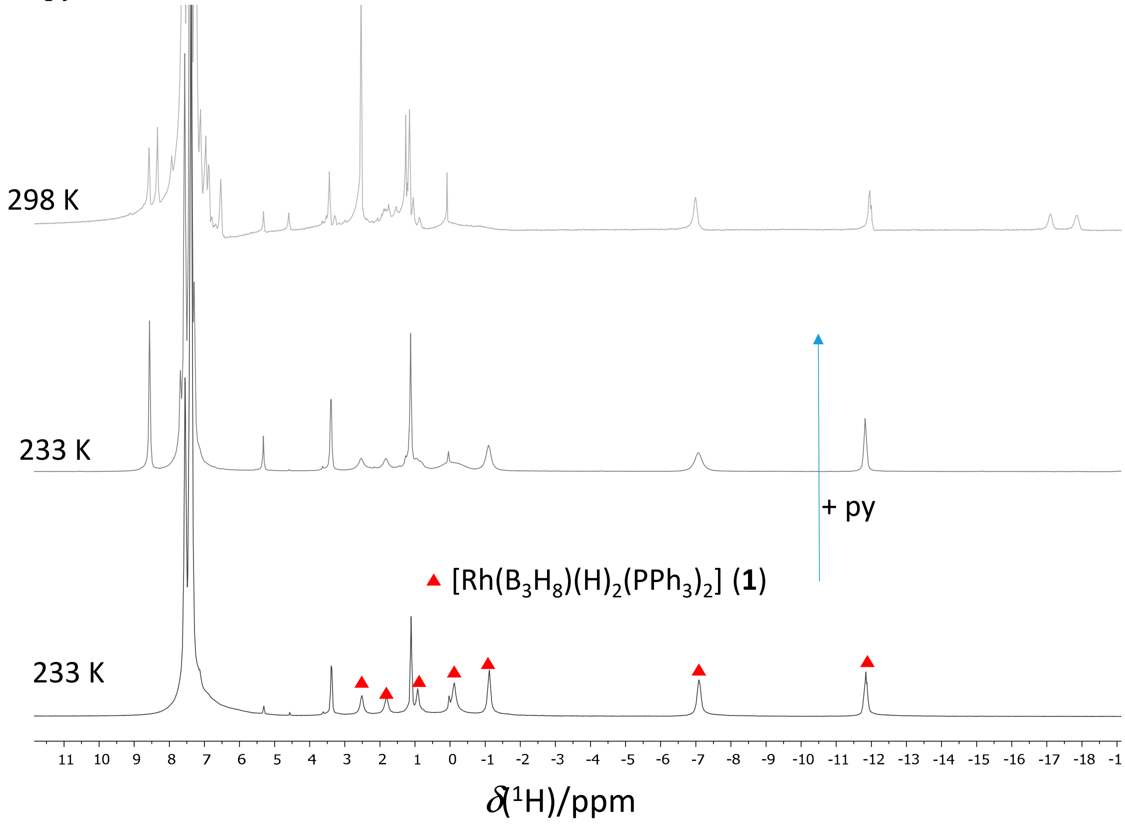

Figure S9 ^{1}H-{^{11}B} NMR spectra from the reaction between [Rh(^{2}-B_3H_8)(H)$_2$(PPh$_3$)$_2$] (**1**) and pyridine, in dichloromethane.

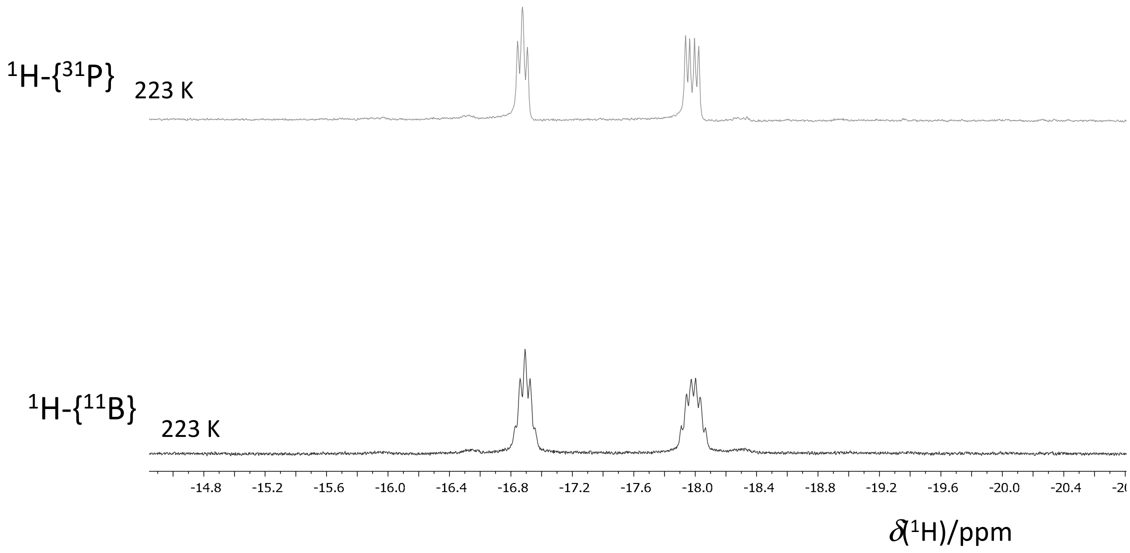

Figure S10 Proton NMR spectra signals from the reaction between [Rh(^{2}-B_3H_8)$(H)_2$$(PPh_3)_2$] (**1**) and pyridine, in dichloromethane.

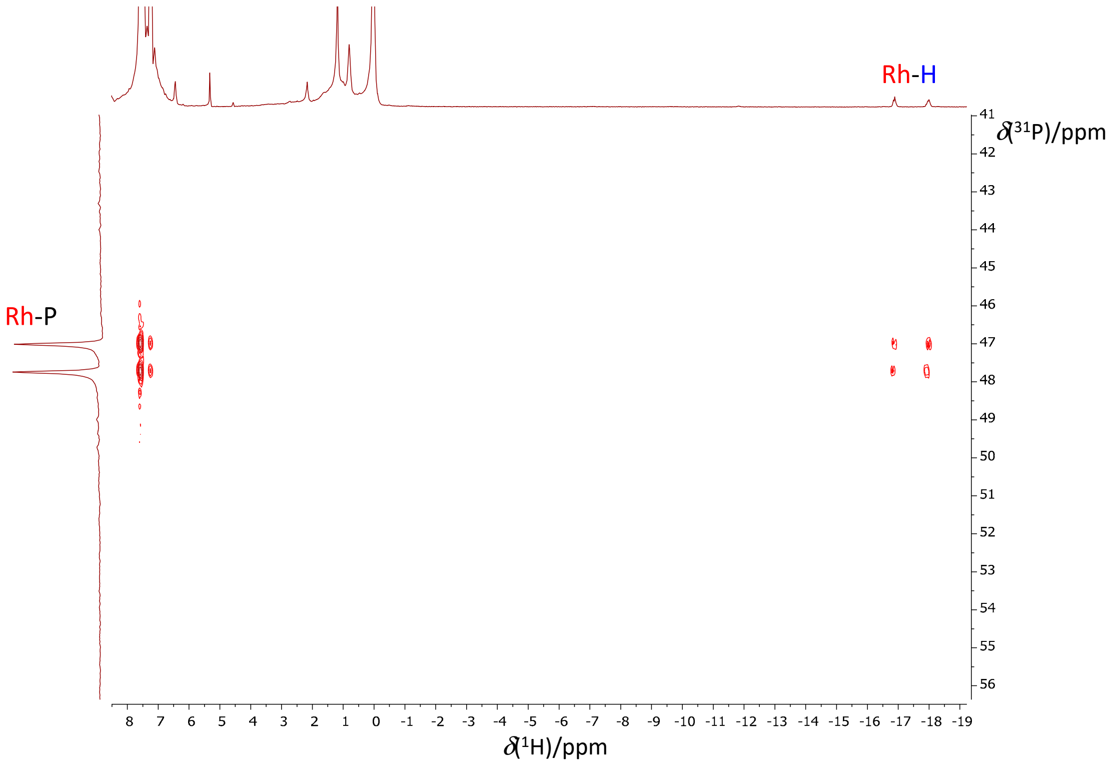

Figure S11 ^{31}P-^{1}H-HMBC NMR spectrum from the reaction between [Rh(^{2}-B_3H_8)$(H)_2$$(PPh_3)_2$] (**1**) and pyridine, in dichloromethane.

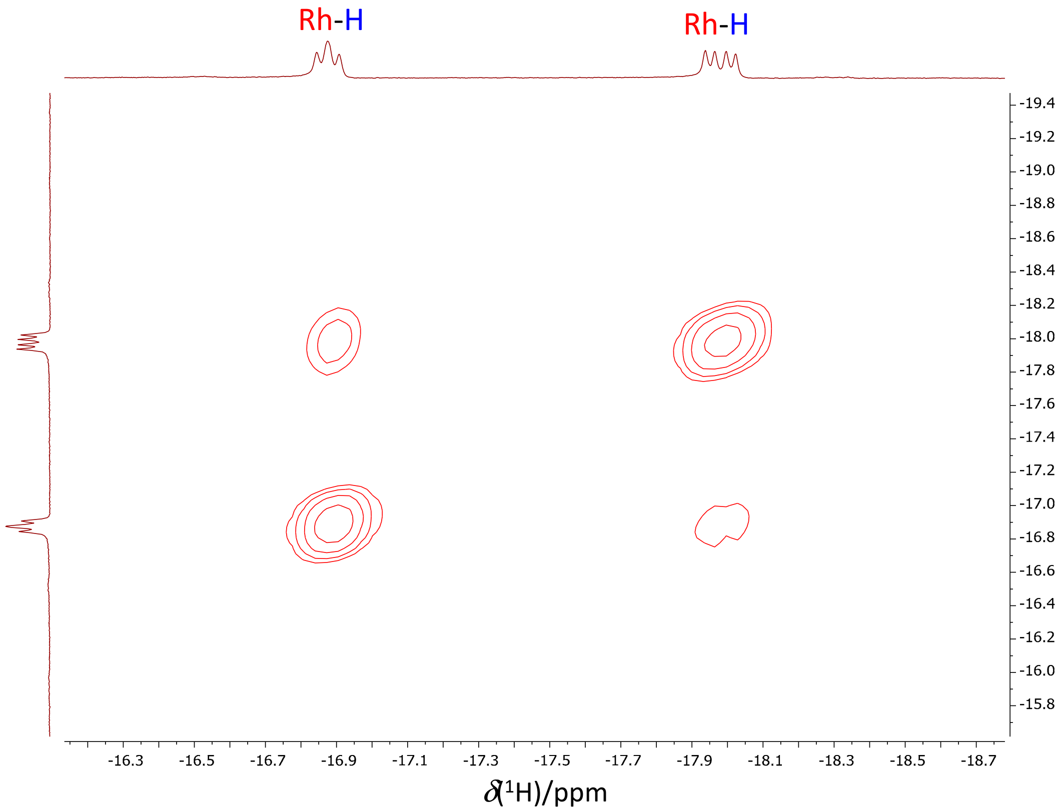

Figure S12 ^{1}H-^{1}H-COSY NMR spectrum, in the hydride region, from the reaction between [Rh(^{2}-B_3H_8)$(H)_2(PPh_3)_2$] (**1**) and pyridine, in dichloromethane.

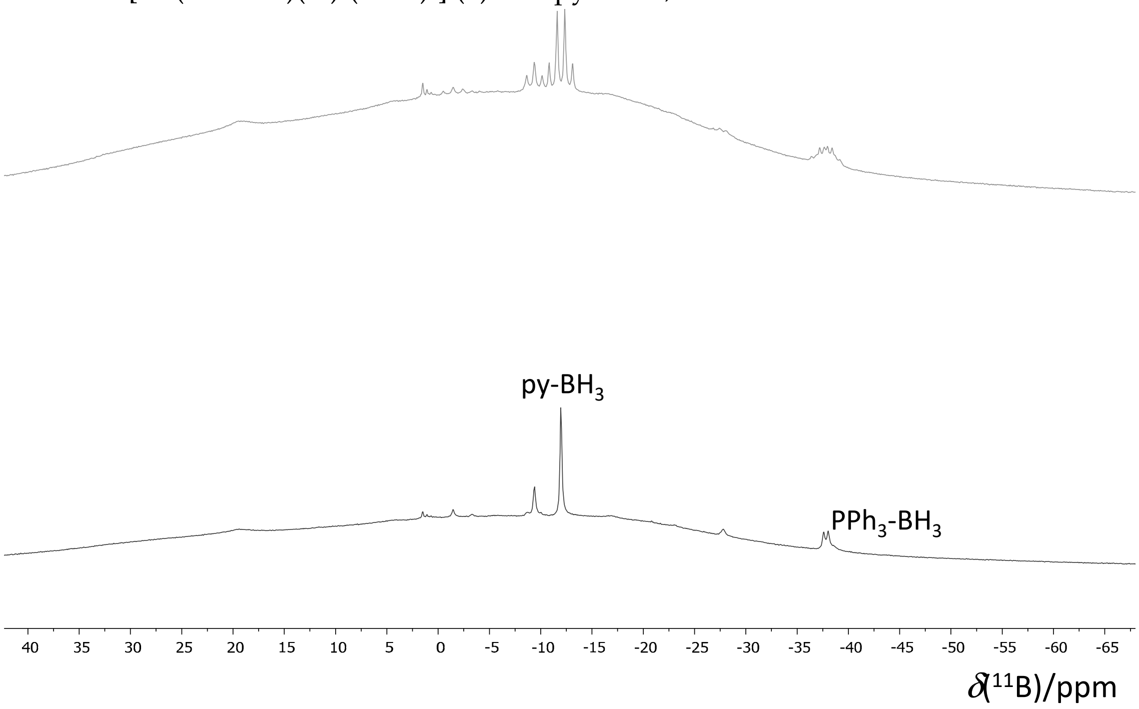

Figure S13 ^{11}B-{^{1}H} NMR spectra from the reaction between [Rh(^{2}-B_3H_8)$(H)_2(PPh_3)_2$] (**1**) and pyridine, in dichloromethane.

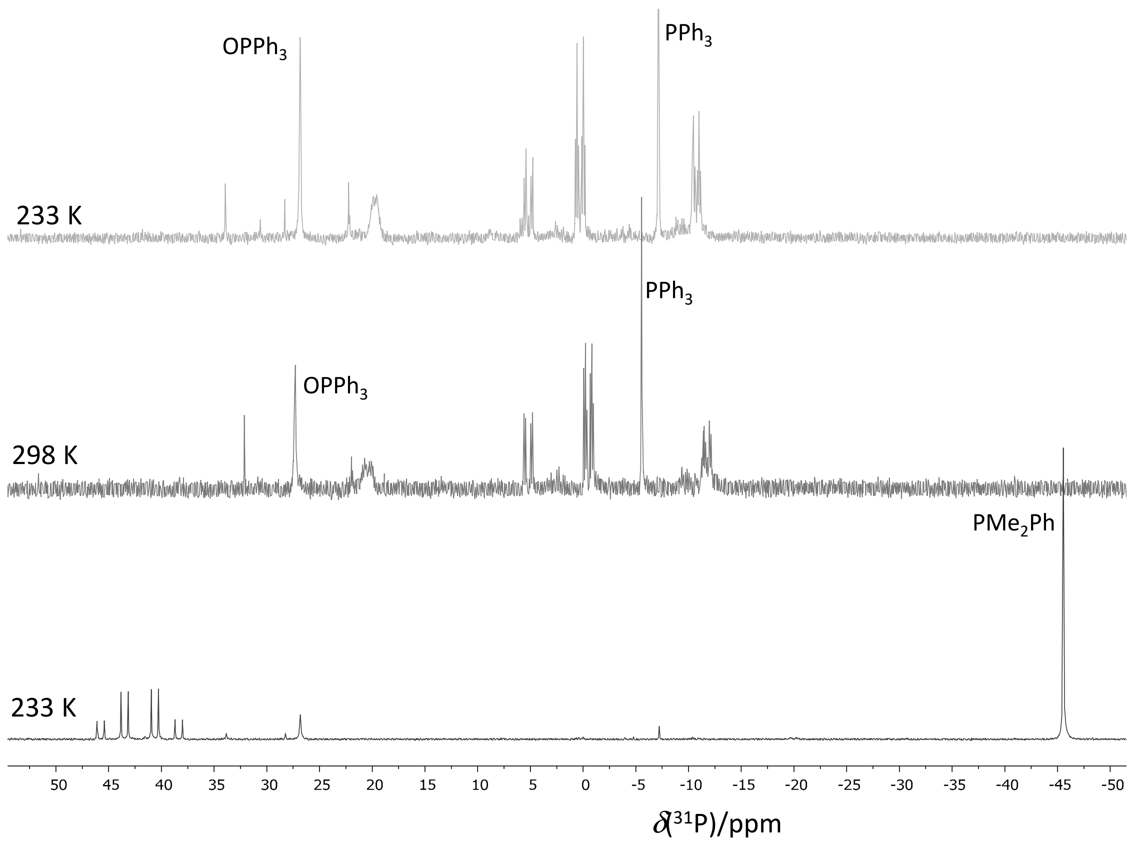

Figure S14 ^{31}P-{^{1}H} NMR spectra that correspond to the reaction between [Rh(^{2}-B_3H_8)$(H)_2(PPh_3)_2$] (**1**) and PMe_2Ph: upon addition of PMe2Ph at 233 K (bottom); after increasing the reaction mixture temperature at 298 K; and at 233 K, in dichloromethane.

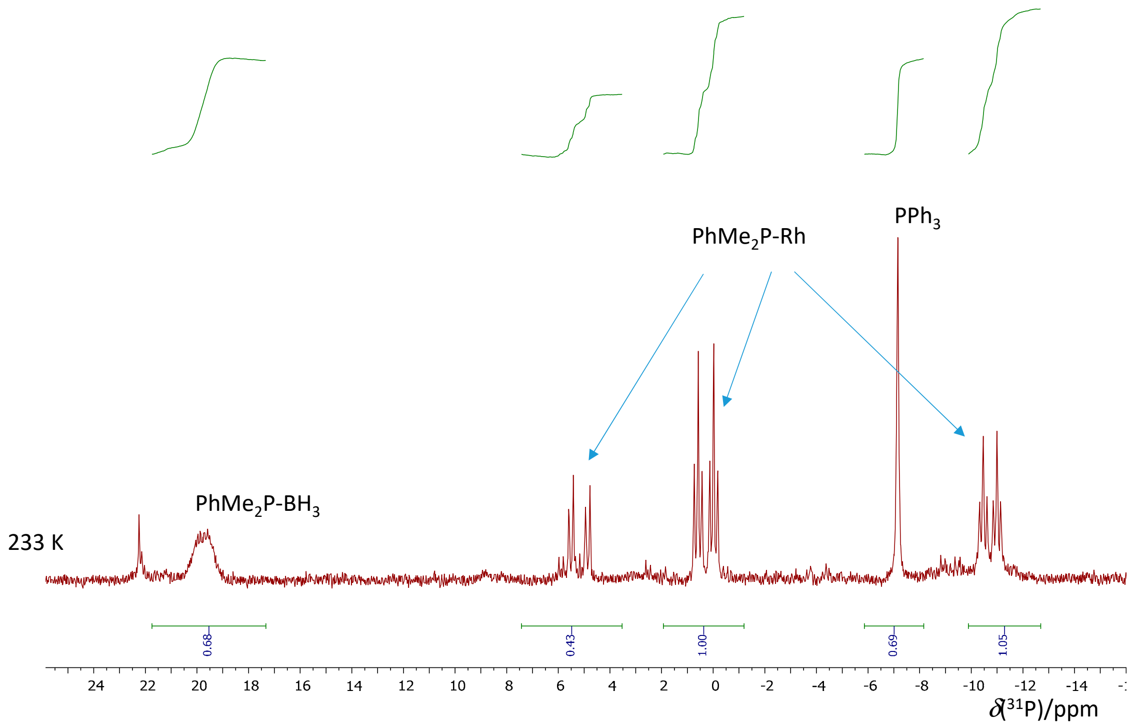

Figure S15 ^{31}P-{^{1}H} NMR spectrum from the reaction between [Rh(^{2}-B_3H_8)(H)$_2$(PPh_3)$_2$] (**1**) and PMe_2Ph, at 233 K, in dichloromethane (upper spectrum in Figure S14).

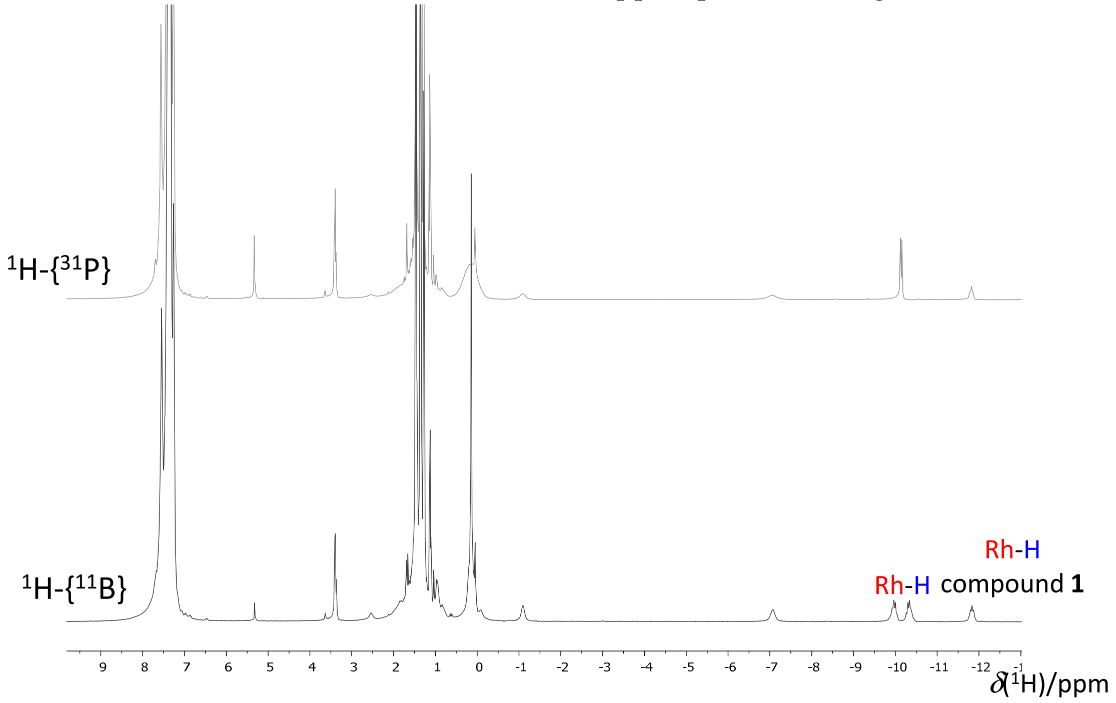

Figure S16 Proton NMR spectra from the reaction between [Rh(^{2}-B_3H_8)(H)$_2$(PPh_3)$_2$] (**1**) and PMe_2Ph, at 233 K, in dichloromethane.

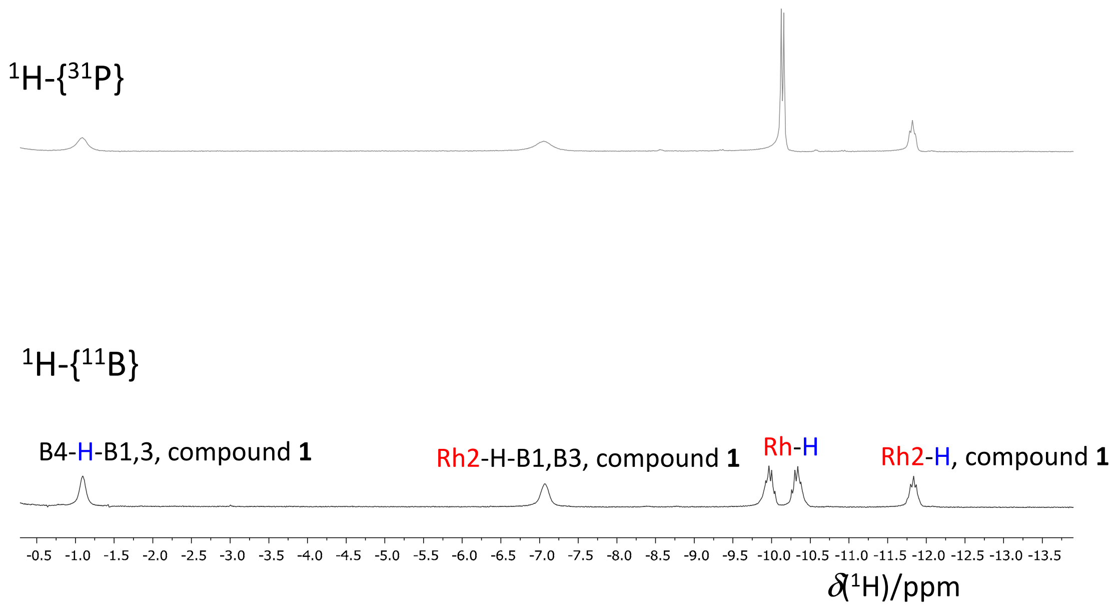

Figure S17 Proton NMR spectra from the reaction between [Rh(^{2}-B_3H_8)(H)$_2$(PPh_3)$_2$] (**1**) and PMe_2Ph, at 233 K, in the negative region (full spectra in Figure S16).

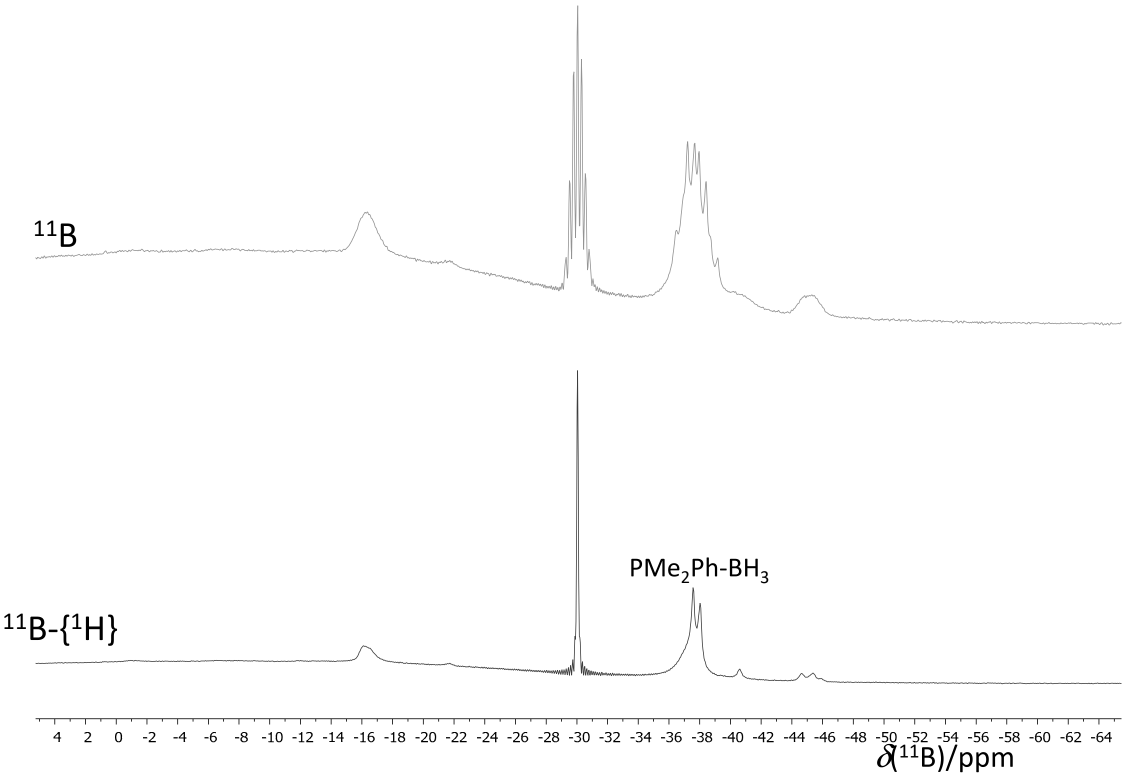

Figure S18 Boron-11 NMR spectra from the reaction between $[Rh(\ ^{2}\text{-}B_3H_8)(H)_2(PPh_3)_2]$ (**1**) and PMe_2Ph, at 29 K, in dichloromethane-d^2.

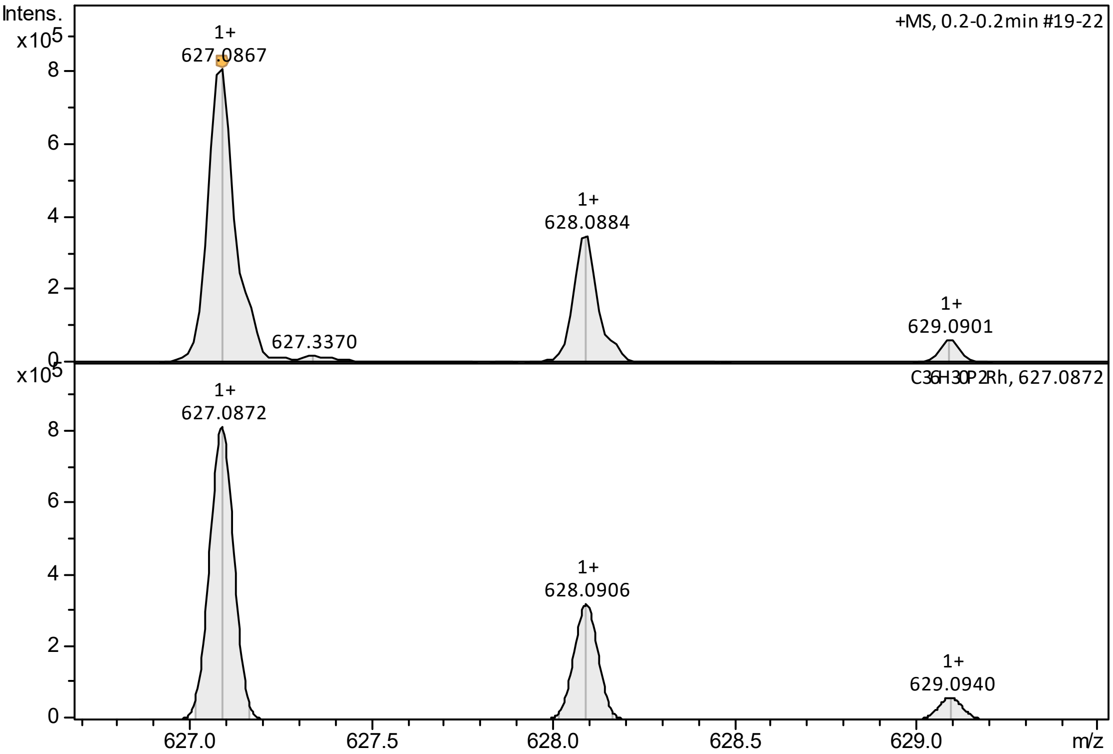

Figure S19 Mass spectrum for **1**: Experimental (upper); theoretical (bottom). The pattern corresponds to the $[Rh(PPh_3)_2]^+$ ion.

Calculation of free energy of activation by the coalescence temperature:

$\Delta G_A^{\ddagger}$ = 4.57 T_c (10.62 + log (X/2π(1-ΔP)) + log (T_c/δν) = 17 kcal/mol

$\Delta G_B^{\ddagger}$ = 4.57 T_c (10.62 + log (X/2π(1+ΔP)) + log (T_c/δν) = 16 kcal/mol

T_c = 300 K

δν (chemical shift difference between the two signals) = 1.83 - (-1.09) = 2.92

ΔP (population site difference) = 2/3 - 1/3 = 0.33

log (X/2π(1-ΔP) = –0.15

log (X/2π(1+ΔP) = –0.60

Based on the treatment by Shanan-Atidi and Bar-Eli (*J. Phy. Chem*. **1970**, *74*, 961), and Egan and Mislow (Thesis Princeton University, 1971).

Table S1. B3LYP/6-31+G(d,p) calculated Cartesian coordinates for PH_3-ligated model compound [Rh(^{2}-$B_3H_8(H)_2(PH_3)_2$]

Atom	x	y	z
Rh	-0.15842700	-0.45213800	-0.00002700
H	-0.42290300	-1.62051800	1.02066000
H	-0.42298400	-1.62060500	-1.02060100
B	0.16110500	1.87046800	0.89370600
B	0.16110100	1.87048400	-0.89358600
B	1.59839300	2.47927100	-0.00000500
H	0.16789500	0.70776700	1.42830700
H	1.21233600	2.29083500	1.42346200
H	-0.73676200	2.52545300	1.33864800
H	2.57618200	1.77816900	0.00036800
H	1.78365500	3.66180700	-0.00024200
H	1.21265900	2.29030300	-1.42328300
H	-0.73605900	2.52607400	-1.33906900
H	0.16765600	0.70784300	-1.42835200
P	2.04803900	-1.17169500	0.00001500
P	-2.44782800	-0.14076000	0.00002000
H	2.88779900	-0.81174700	1.07400700
H	2.88775800	-0.81210200	-1.07413100
H	2.27012600	-2.56361500	0.00023600
H	-3.01783300	0.57225600	1.07501800
H	-3.01797900	0.57168800	-1.07527800
H	-3.28852000	-1.27167800	0.00036300
